# Autoimmune Encephalitis in Pregnancy: A Case Report

**DOI:** 10.7759/cureus.98289

**Published:** 2025-12-02

**Authors:** Nikolaos Antonakopoulos, Panagiota Tzela, Leonidas Antonakis, Nikolaos Thanatsis, Georgios Adonakis

**Affiliations:** 1 Department of Obstetrics and Gynecology, School of Health Sciences, University of Patras, Patras, GRC; 2 Department of Midwifery, School of Health and Care Sciences, University of West Attica, Athens, GRC

**Keywords:** antibody-negative encephalitis, autoimmune encephalitis, cognitive deficits, covid-19, neurological disorders, pregnancy, seizures

## Abstract

Autoimmune encephalitis is a rare and heterogeneous group of inflammatory disorders of the central nervous system, occasionally occurring during pregnancy and posing diagnostic and therapeutic challenges. Pregnancy-related physiological changes and the need to avoid potentially teratogenic treatments often delay diagnosis and intervention. We report the case of a 27-week primigravida who presented with acute mental status deterioration. On admission, she was hemodynamically stable but exhibited a Glasgow Coma Scale (GCS) score of 10/15. Fetal bradycardia prompted an emergency cesarean section. Neuroimaging revealed mild temporal and parietal lobe edema, while cerebrospinal fluid analysis showed borderline lymphocytic pleocytosis without infectious or autoimmune antibodies. Despite the absence of specific immunomodulatory therapy, the patient improved gradually with supportive and antiepileptic treatment (levetiracetam and phenytoin) and was discharged in good condition after two weeks. Compared with published reports, this case was atypical due to its onset in the late second trimester, minimal neuroimaging findings, and negative antibody profile. A recent COVID-19 infection preceding symptom onset was also unique among documented cases. The favorable outcome without immunotherapy suggests that some seronegative, mild presentations may follow a self-limited course. Our case adds to the growing evidence that autoimmune encephalitis in pregnancy can present with atypical and antibody-negative features that mimic other obstetric complications, such as eclampsia. Prompt multidisciplinary assessment, careful exclusion of alternative diagnoses, and close neurological follow-up are critical to optimizing maternal and fetal outcomes.

## Introduction

Autoimmune encephalitis is a heterogeneous group of autoimmune‐mediated disorders involving the central nervous system (CNS). The estimated prevalence is 14 cases per 100,000, and the median age of patients is 43, based on a large US study [1]. Symptoms mainly include cognitive and/or behavioral deficits, seizures, and movement disorders. In most cases, a prodromal viral infection precedes [2]. The disease may also be the manifestation of an underlying neoplasia, such as breast, ovarian, testicular, or lung cancer, as well as Hodgkin's lymphoma [3,4]. The diagnosis is based on clinical, laboratory, and magnetic resonance imaging (MRI) findings. Autoantibodies against antigens localized in distinct neuroglial components are considered the pathophysiological origin of the disease; however, in up to 20% of cases, antibodies are not detected in serum or cerebrospinal fluid (CSF), making differential diagnosis even more challenging [5].

Autoimmune encephalitis is possible during pregnancy [6,7]. Diagnosis and treatment during pregnancy are challenging due to the possible teratogenic effects of certain diagnostic procedures and therapeutic approaches, especially in the first trimester [8-10]. Even more, any atypical appearance of the disease can raise suspicion for other, more common pregnancy-related conditions, and usually the diagnosis is made late by exclusion. We present a case of autoimmune encephalitis of atypical appearance during pregnancy, which was initially considered eclampsia. An updated review of the current literature is also provided.

## Case presentation

The patient was a 27-week primigravida (G1P0) admitted to our tertiary care university hospital due to an acute deterioration in mental status. The exact time of symptoms' onset was difficult to determine since the woman was home alone for approximately eight hours, but her husband detected the change in her mental status as soon as he returned home and brought her to the hospital. A multidisciplinary team comprising anesthesiologists, neurologists, and obstetricians was immediately assembled for evaluation.

On admission, she was hemodynamically stable with satisfactory oxygen saturation, although mild tachycardia was noted. Her Glasgow Coma Scale (GCS) score was 10/15. Otherwise, no significant findings were present during her physical examination or laboratory testing. Cranial nerve testing was unremarkable: pupils were equal and reactive, extraocular movements were full, facial strength and sensation were normal, hearing was intact, and gag reflex, tongue movements, and shoulder shrugging were symmetric. Motor examination revealed normal bulk and tone, without fasciculations or involuntary movements. Strength was 5/5 across all major muscle groups in the upper and lower extremities. Sensory testing showed intact light touch, pinprick, vibration, and proprioception bilaterally. Deep tendon reflexes were 2+ and symmetric throughout, with flexor plantar responses and no clonus. Coordination was normal on finger-to-nose testing. Concerning her medical history, the only remarkable finding was a mild COVID-19 infection two weeks preceding symptoms.

Fetal assessment demonstrated persistent severe fetal bradycardia (fetal heart rate (FHR) of 60-80 bpm), prompting an uncomplicated emergency cesarean section. Apgar scores of 2 and 4 at the first and fifth minutes confirmed the suspected severe fetal compromise. Unfortunately, the neonate passed away several days later due to extreme prematurity complications.

Postoperatively, brain computed tomography (CT) revealed no evidence of ischemia, hemorrhage, or cerebral venous sinus thrombosis. Lumbar puncture showed normal CSF parameters, except for borderline lymphocytic predominance (Table 1). Basic metabolic and infectious workups, including electrolytes, liver function tests, thyroid antibodies, and viral PCRs, were all negative. Levetiracetam therapy (500 mg twice daily) was initiated after the initial workups (brain CT, lumbar puncture, and basic metabolic and infectious workups) to prevent seizures in case the woman had an atypical form of nonconvulsive status epilepticus or seizures at home prior to her admission, since she was alone, and this possibility could not be excluded. Additionally, neurologists believe it may suppress some aspects of neuroinflammation. Even more, acyclovir 750 mg/8 h was initiated since a minority of patients with all-cause encephalitis exhibit absence of pleocytosis, and acyclovir administration should not be delayed. Blood pressure was always below 140/90 mmHg, and transthoracic echocardiography findings were unremarkable. The woman's blood pressure, proteinuria, and blood count/liver function test results never exceeded the cutoff threshold for pre-eclampsia diagnosis.

**Table 1:** Patient's cerebrospinal fluid parameters

Parameter	Patient Values	Reference Range
Appearance	Clear and colorless	Clear and colorless
Specific gravity	1.008	1.006–1.008
Protein	50 mg/dL	15–60 mg/dL
Glucose	62 mg/100 mL	50–80 mg/100 mL
White blood cell (WBC) count	5 per μL	0–5 per μL
Lymphocytes	4 per μL	0–3 per μL
Monocytes	1 per μL	0–2 per μL
Lactate	19.6 mg per dL	11.7–21.6 mg per dL
Bacteria	0	0

**Figure 1:**
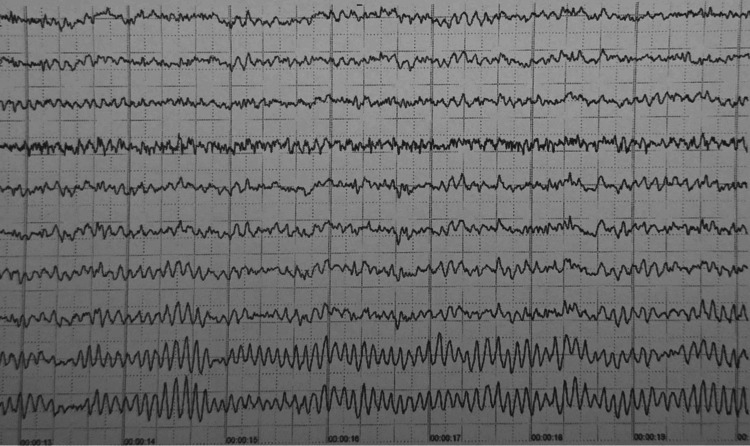
Patient's normal EEG. The recording shows a well-organized, continuous, and symmetric background rhythm, consisting of 8-10 Hz alpha activity, with preserved posterior dominance, normal variability in amplitude and frequency across channels, low-amplitude beta frequencies, and normal sleep-wake features. No epileptiform discharges, such as spikes or sharp waves, no focal abnormalities, and no ictal patterns are seen.

**Figure 2:**
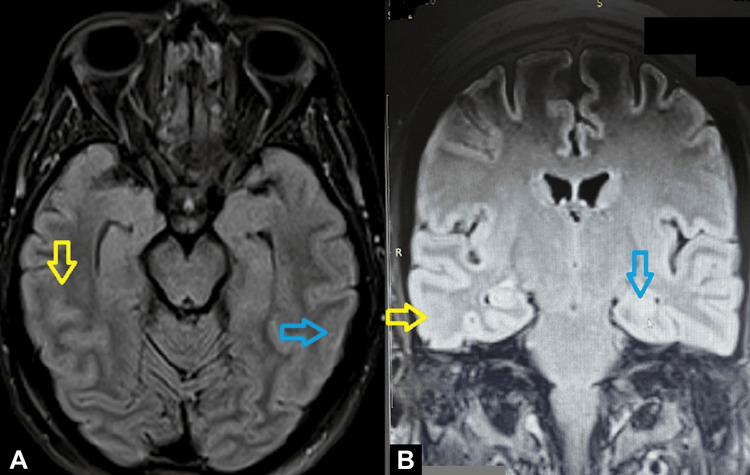
(A, B) Patient's magnetic resonance imaging (MRI). Yellow arrows demonstrate mild edema/unclarities of the right temporal lobe. Blue arrows demonstrate mild edema/unclarities of the left temporal lobe. MRI descriptions may be vague when abnormalities are subtle, evolving, affected by technical limitations, or when the underlying disease, such as encephalitis, frequently produces non-specific or even normal imaging findings, especially early in its course.

Given the post-delivery persistent neurological impairment (GCS 8/15), phenytoin (15 mg/kg infusion) was added to cover the possibility of non-convulsive status epilepticus. An electroencephalogram (EEG) was available and performed the next day, and it was given as normal, so phenytoin was gradually discontinued (Figure 1). MRI later demonstrated mild edema of the temporal and parietal lobes, predominantly on the right side (Figure 2). A presumptive diagnosis of autoimmune encephalitis was established, although the presentation was atypical. The patient's condition gradually improved, and she was discharged after two weeks with instructions for regular neurological follow-up.

Written informed consent for the publication of this case report was obtained from the patient.

## Discussion

According to the literature, 43% of cases of autoimmune encephalitis during pregnancy occurred in the first trimester, 41% in the second, and only 16% in the third [11]. In comparison to our case at 27 weeks' gestation, less than 20% of cases manifest at or after this pregnancy time point. To our knowledge, our case is the only one in the literature of a pregnancy with a COVID-19 infection preceding symptom onset. In our case, the possibility of acute COVID-19 encephalitis was considered unlikely, not only because the woman had recovered from COVID-19 several days earlier, but also because acute encephalitis is an uncommon complication of COVID-19 [12]. Nevertheless, MRI failed to show findings compatible with acute COVID-19 infection of the brain. On the other hand, COVID-19 encephalitis, when present, results in significant morbidity and mortality [12].

A prodromal viral syndrome preceding autoimmune encephalitis is well described, particularly following herpes simplex virus (HSV) encephalitis. Several complementary mechanisms likely explain this association [13-16]: virus-induced neuronal injury with release of sequestered antigens and subsequent epitope spreading; molecular mimicry leading to cross-reactive antibodies (for example, anti-NMDAR after HSV); bystander activation in a pro-inflammatory environment; and blood-brain barrier disruption that permits peripheral autoreactive lymphocytes and antibodies to access CNS targets. In some patients, viral persistence or incomplete clearance may sustain immune activation and precipitate a secondary autoimmune phase. While animal and human data support these pathways, the precise sequence and relative contribution of each mechanism vary between patients and disease contexts and remain an area of active investigation. The link with COVID-19 is speculative, and this case report triggers further epidemiologic research to support this hypothesis.

The most frequent symptoms in the literature were psychiatric manifestations, in 2/3 of the patients, such as visual hallucinations and acute psychosis. Seizures were also frequent (41% of cases), followed by headache in 1/3 of the patients. Cognitive impairment, like in our case, was noted in only 20% of cases [11]. Half of the patients were admitted to the Intensive Care Unit due to the severity of symptoms. Our case was among the more severe reported cases, given a GCS score of 10.

CSF disturbances in encephalitis typically include an increased white blood cell count (pleocytosis) with a lymphocytic predominance, normal to moderately elevated protein levels, and normal glucose levels [12]. Pleocytosis is not always present and can be absent early on or in specific types, particularly in autoimmune encephalitis or in immunocompromised individuals, such as in pregnancy [12,17]. Since 25.3% of patients with all-cause encephalitis exhibit absence of pleocytosis, acyclovir initiation should not be delayed in the absence of pleocytosis in patients with suspected encephalitis [18]. Specific findings can vary, so a comprehensive analysis of the CSF is important, and other diagnostic tests like MRI and EEG are crucial for a definitive diagnosis [19].

Serum or CSF autoantibodies were detected in 2/3 of the cases. The most frequent autoantibodies detected were against the anti‐NMDAr, in 3/4 of the cases [11]. EEG analysis was normal in 43% of cases; diffuse theta/delta slowing was reported as the main pathological finding, whereas epileptiform discharges were detected in only 14% of cases [11].

MRI findings were present in half of the patients. The typical change is a hyperintense signal on T2‐weighted and T2‐FLAIR sequences, mostly in the temporal region [11,19]. Interestingly, an MRI can be initially normal, with a repeat MRI subsequently showing abnormalities [17]. Mesial temporal lobe sclerosis may be a late finding, especially among autoimmune limbic encephalitides [20]. Neuroimaging findings in autoimmune encephalitis vary widely. Limbic involvement remains the most characteristic pattern, demonstrating T2/FLAIR hyperintensities within the mesial temporal structures, frequently without associated diffusion restriction or contrast enhancement. These changes may be unilateral or bilateral and are usually non-specific in the early stages, sometimes appearing only as subtle cortical or subcortical signal alterations. Notably, up to one-third of patients with autoimmune encephalitis show normal or near-normal MRI findings, particularly in seronegative or non-limbic variants, which underscores why radiologic descriptions may be vague or minimally abnormal. In this context, even mild or asymmetric signal changes, such as the subtle temporal-parietal edema observed in our case, can be compatible with autoimmune encephalitis, especially when correlated with the clinical presentation and exclusion of alternative causes.

Most patients in the literature received immunomodulant treatment, such as corticosteroids, intravenous immunoglobulins, or plasma exchange. Second-line immunosuppressive treatments, such as rituximab, cyclophosphamide, and azathioprine, were administered in a minority of patients [11]. Anti-seizure treatment was reported in 61% of patients. The first-line treatment consisted of the administration of benzodiazepines in combination with further antiseizure medications, such as levetiracetam, phenytoin, and valproic acid [11]. Antipsychotic treatment was reported in 12% of cases, with haloperidol being the most frequently employed medication, followed by olanzapine, quetiapine, and tiapride [11].

Most of the cases were delivered via cesarean section. Hopefully, the mortality rate was low, approximately 6%. First-line immunotherapy (single or combined) was generally employed, while second-line immunotherapy was administered to a minority of patients. Levetiracetam was the most used antiseizure medication. Half of the patients recovered completely, but the remaining cases showed persistent deficits. Moderate to severe motor deficits were improved after a rehabilitation program. A logistic regression performed to identify predictors of maternal outcome failed to show any significant variables [11].

Concerning fetal outcome, most of the pregnancies progressed unremarkably and led to normal delivery, although premature births were reported in some cases. However, in 1/4 of cases, fetal death was reported. One neonatal death shortly after due to severe neonatal complications was described [11]. Unfortunately, this was the fetal outcome in our case as well. Of course, the neonatal compromise in our case was mainly due to in utero fetal compromise. A logistic regression performed to identify predictors of fetal outcome failed to show any significant variables [11].

Compared with previously published cases [11,21], our patient's presentation was atypical in several respects. First, the onset occurred in the late second trimester, a period less frequently reported in autoimmune encephalitis during pregnancy, whereas most cases manifest in the first or early second trimester. Second, our patient had a recent history of COVID-19 infection preceding symptom onset, a feature rarely documented in the literature and representing a possible, though unproven, immunological trigger. Neuroimaging findings in our case were subtle compared with the more extensive temporal hyperintensities typically described in anti-NMDAR or limbic encephalitis. Additionally, CSF analysis showed only borderline lymphocytic pleocytosis and no evidence of infectious or autoimmune markers, emphasizing the diagnostic complexity in antibody-negative cases.

Therapeutically, the patient received supportive and antiepileptic management with levetiracetam and phenytoin, without immunomodulatory therapy, yet demonstrated gradual neurological recovery. This favorable outcome, despite the absence of targeted immunotherapy, highlights the variable disease course and the potential role of spontaneous recovery or limited autoimmune activity in some atypical cases. A large retrospective study of 182 patients found that 80% achieved "good recovery" at a median of four months after the acute attack [22]. Our report thus underscores that autoimmune encephalitis in pregnancy can present with minimal MRI abnormalities, negative serology, and a favorable prognosis under supportive treatment alone, diverging from the more severe, antibody-positive cases described in the literature.

## Conclusions

Autoimmune encephalitis during pregnancy remains a rare and diagnostically challenging condition, particularly when the presentation is atypical or seronegative. Our case adds to the growing evidence that autoimmune encephalitis may occur in the absence of specific antibody detection and with only subtle radiologic findings. Early multidisciplinary evaluation, exclusion of pregnancy-related mimics such as eclampsia, and timely supportive care are essential for optimizing maternal outcomes. Although most reported cases require immunomodulatory therapy, our patient's gradual neurological improvement without it suggests that disease severity and immune activation may vary substantially among individuals. Given the potential for significant maternal and fetal morbidity, heightened clinical suspicion, close peripartum monitoring, and long-term neurological follow-up are warranted. Further studies and accumulated case data are needed to clarify prognostic factors, optimal therapeutic timing, and the role of pregnancy-related immune modulation in the pathogenesis of autoimmune encephalitis.
